# Introducing the PLOS special collection of economic cases for NCD prevention and control: A global perspective

**DOI:** 10.1371/journal.pone.0228564

**Published:** 2020-02-06

**Authors:** Rachel A. Nugent, Muhammad Jami Husain, Deliana Kostova, Frank Chaloupka

**Affiliations:** 1 Global NCDs, RTI International, Seattle, Washington State, United States of America; 2 Department of Global Health, University of Washington, Seattle, Washington State, United States of America; 3 Division of Global Health Protection, Centers for Disease Control and Prevention, Atlanta, Georgia, United States of America; 4 University of Illinois, Chicago, Illinois, United States of America; Osakidetza Basque Health Service, SPAIN

## Abstract

Noncommunicable diseases (NCDs), such as heart disease, cancer, diabetes, and chronic respiratory disease, are responsible for seven out of every 10 deaths worldwide. While NCDs are associated with aging in high-income countries, this representation is often misleading. Over one-third of the 41 million annual deaths from NCDs occur prematurely, defined as under 70 years of age. Most of those deaths occur in low- and middle-income countries (LMICs) where surveillance, treatment, and care of NCDs are often inadequate. In addition to high health and social costs, the economic costs imposed by such high numbers of excess early deaths impede economic development and contribute to global and national inequity. In higher-income countries, NCDs and their risks continue to push health care costs higher. The burden of NCDs is strongly intertwined with economic conditions for good and for harm. Understanding the multiple ways they are connected–through risk factor exposures, access to quality health care, and financial protection among others–will determine which countries are able to improve the healthy longevity of their populations and slow growth in health expenditure particularly in the face of aging populations. The aim of this Special Collection is to provide new evidence to spur those actions.

## Introduction

Noncommunicable diseases (NCDs), such as heart disease, cancer, diabetes, and chronic respiratory disease, are responsible for seven out of every 10 deaths worldwide [1]. While NCDs are associated with aging in high-income countries, this representation is often misleading. Over one-third of the 41 million annual deaths from NCDs occur prematurely, defined as under 70 years of age. Most of those deaths occur in low- and middle-income countries (LMICs) where surveillance, treatment, and care of NCDs are often inadequate. Health risks commonly seen in higher-income countries, such as tobacco consumption, heavy use of alcohol, and unhealthy diet, are now common in LMICs, driven both by economic progress and the encroachment of transnational companies marketing those products into all facets of society. In addition to high health and social costs, the economic costs imposed by such high numbers of excess early deaths impede economic development and contribute to global and national inequity. In higher-income countries, NCDs and their risks continue to push health care costs higher. Countries seek new ways to slow growth in health expenditure particularly in the face of aging populations. The aim of this Special Collection is to provide new evidence to spur those actions.

The International NCD Economics Research Network, sponsored by the US Centers for Disease Control since 2017, launched this PLOS Special Collection in January 2019 at the Prince Mahidol Award Conference (PMAC). The theme of the conference was “Political Economy of NCDs: A Whole of Society Approach.” The Special Collection was well suited to the theme of political economy as it explores the case for investing in NCD control in developing countries, household economic impacts from NCDs, and a range of risk factors that contribute to NCDs. The sixteen articles in the Collection offer an economic rationale for scaling-up interventions to address NCDs, provide deeper insight into policy options for preventing NCDs, and help us understand the household impacts of NCDs such as shifts in consumption patterns. The Special Collection covers a range of economic methods including investment cases for NCD interventions, costs and cost-effectiveness of NCD risk reduction policies and treatment protocols, and distributional impacts of NCDs and control interventions. The articles have been contributed by scholars from around the world seeking to build a stronger evidence base for action on NCDs, a neglected area of global health.

The articles in this collection highlight the economic burden of NCDs and the impact of NCD prevention and control programs globally under three themes: 1) national economic analysis, including investment cases, 2) household resource allocation, and 3) policy interventions addressing NCD risk factors. The studies contained within this Collection use a range of economic methods and explore multiple economic outcomes. We offer brief summaries of each paper followed by a discussion of the implications of the Special Collection and the recommended directions for future research on the economics of NCDs. Table A in S1 Appendix lists the key features of the articles clustered within the three main themes.

### Theme 1: National analyses including NCD investment cases and economic evaluation

Economic evaluations of NCDs are essential to understanding how these diseases can shape society. NCDs, which are associated with high productivity losses and healthcare costs, can strain economies with limited healthcare systems, undermine social and economic development, and dramatically affect a country’s health security and stability. A growing literature using economic modeling to quantify the results of investments in NCD prevention and control shows that millions of premature deaths can be averted and billions in economic output gained [1–3]. The articles in Theme 1 of this Collection examine the costs, cost-effectiveness and return on investment of NCD care. They demonstrate that NCDs are expensive but that many cost-effective prevention and control strategies are available to countries. However, returns on investment in such strategies vary by country, depending on the initial levels of disease prevalence, prevention and screening, and treatment interventions in a country. Other factors that affect results in these economic analyses are the costs of the health system interventions and the medical and non-medical costs of illness. Thus, countries could draw guidance from economic evaluations done elsewhere but base their own planning and priority-setting on analysis that uses local data and health conditions.

Chen et al. assessed the extent to which NCDs would affect U.S. productive capacity from 2015–2050 using the “EPIC-Health” macroeconomic model to calculate the resulting economic burden in terms of foregone GDP [4]. EPIC-Health incorporates human capital into the production function along with the impacts of reduced productivity on savings and investment. It also captures more dynamic flows between capital and labor and encompasses more health outcomes than earlier versions of the model, including mental health. The model accounts for the impact of NCDs on economic growth including mortality, morbidity, and treatment costs. It incorporates age-specific human capital measures such as education-related productivity over time. Mental health conditions and cardiovascular diseases impose the highest economic burdens in the U.S., followed by cancer, diabetes, and chronic respiratory diseases. Chen et al. estimated a total loss of USD 94.9 trillion, or USD 265,000 per capita (in constant 2010 USD) attributable to NCDs in 2015–2050, which corresponds to an annual tax rate of 10.8% on aggregate income.

A simpler modeling approach to measuring the economic impacts of NCDs and the return on investment (ROI) for NCD control is used in Hutchinson et al.’s case study for Jamaica [5]. The authors combined a quantitative analysis of the benefits and costs of NCD control in Jamaica with a qualitative political economy analysis of government and stakeholder readiness for change. The quantitative model used the Spectrum-based OneHealth Tool, developed by the World Health Organization and other UN agencies, to examine the ROI for a package of selected prevention and treatment services. The model has regularly updated demographic, epidemiologic, and cost inputs developed with guidance from a UN inter-agency group. The One Health Tool was populated with locally-relevant data through the support of technical experts at the Jamaican Ministry of Health and other public agencies in Jamaica. Results were disseminated widely across stakeholders in the country including the media. The case study offers a real-world experience of how to employ simple economic analysis to illustrate a central point about NCDs and their impacts on economies.

Cecchini used a U.S. dataset on health expenditures to project the effect of rising obesity on health care utilization and costs [6]. The two-step analysis entailed a logistic regression to estimate the probability of future obesity among normal-weight and obese people and a generalized linear model to examine health expenditure outcomes among those who access services. Healthcare services included inpatient care after surgery, inpatient care without surgery, office-based care, outpatient-care, drug prescription, and home healthcare. Results indicate that normal-weight and overweight individuals are expected to have stable trends in the use of future healthcare services, while patients with class II and III obesity would observe increased use. Thus, total healthcare expenditures will increase faster in the obese population than in normal-weight populations.

Yurekli et al. focused their study on a major NCD risk factor: hypertension. The authors examined the economic benefits and costs of reducing hypertension burden due to reduction in population-wide sodium consumption and projected the reduced hypertension prevalence and deaths in Turkey from 2015–2030 [7]. No specific model was used. The authors project hypertension prevalence and future hypertension-associated mortality econometrically based on estimated reductions in salt consumption and consequent reductions in population-level blood pressure. They convert the changed health burden into monetary terms. This provides estimates of the economic benefits of reducing hypertension-associated deaths. Costs of treatment include ambulatory care and medications. In leaving out the productivity and cost of illness impacts, this study calculates the economic impact of reducing hypertension more narrowly than the previously described studies but still results in economically positive returns.

Camacho et al. looked at health expenditure for CVD–this time in Colombia, a middle-income economy [8]. The researchers were able to obtain patient data from HealthCare, a private health insurance database, to calculate average annual costs for CVD patients [8]. No modeling was necessary as direct cost data for NCD care was obtained from a reliable and representative population, making this study a valuable exemplar. This is the first assessment of the cost of healthcare among CVD patients in Colombia and demonstrates a high financial burden.

Subramaniam et al. sought to assess the cost-effectiveness of a risk-stratified approach to medical management of hypertension cardiovascular disease events in Kenya [9]. Using the Kenya 2015 STEPwise survey, the study stratified the population into four risk groups and assessed the cost-effectiveness of care for each group. They employed a macrosimulation model of a cohort of individuals to evaluate CVD risk over the lifetime and found that it was more cost-effective to treat high-risk individuals as compared to treating high- and moderate-risk individuals.

Feigl et al. examined employment-related costs associated with NCDs and their risk factors across OECD-plus countries [10]. Using panel microdata on health and socioeconomic status from 2004–2015 that includes more than 120,000 people in 21 countries, Feigl et al. modeled the short-term impact of BMI, alcohol use, and associated NCDs on employment likelihood, intent to retire early, days of absenteeism, and hours worked per week. Two models were employed: a lagged Poisson regression to examine the effect of health outcomes on labor market variables, and a Zero-inflated Poisson regression to examine absenteeism and hours worked. The results show that NCDs associated with being overweight and alcohol use harmed labor market outcomes, although there were wide differences between men and women. The findings also suggest that having co-morbidities can be an important factor determining hours worked and absenteeism, but the study was not designed to report on those outcomes. The rich dataset points toward important non-health outcomes from behavioral risks related to NCDs.

### Theme 2: NCD impacts on household resource allocation

Households experience NCDs differently than national economies. The aggregation of household economic impacts from NCDs produces the kind of national-level results described above, but decisions within households determine how the impacts are distributed across society. Behavioral and environmental factors determine risk exposures within and across households while policy and health system factors affect awareness and response to those risks. Two studies in the Collection use household income and expenditure data from a lower-middle income country, Bangladesh, to examine the impact of NCDs and their risk factors on other spending.

Husain et al. assessed the crowding-out effect of tobacco consumption in Bangladesh on other purchases in households with and without a tobacco user [11]. They used Seemingly Unrelated Regressions (SUR) to estimate the conditional Engel curves for various expenditures such as food, clothing, and housing. Households that contained a tobacco user allocated less on clothing, housing, education, energy, transportation, and communication than households without tobacco users. Households with tobacco users spent more on food and medical expenditures than households without tobacco users. Based on spending patterns within households, the study suggests that tobacco expenditure has a negative impact on the human capital investment potential at the household level.

Datta et al. assessed the role of NCDs in household resource allocation in Bangladesh [12]. Household NCD status was based on a member of the household having at least one of the six major NCDs: heart disease, hypertension, diabetes, kidney diseases, asthma, and cancer. Unadjusted and regression-adjusted differences in household expenditure shares between NCD and non-NCD households were assessed. Households with members who have NCDs were associated with lower relative expenditure on clothing, housing, hygiene, and energy in all economic groups, but spent more on tobacco and higher-calorie foods such as sugar, beverages, meat, dairy, and fruit. Medical expenditures were 59% higher in NCD households than non-NCD households.

### Theme 3: Policies to reduce NCD risk factors

According to the WHO and the Disease Control Priorities, 3^rd^ Edition, preventive policies are the most cost-effective actions that countries can undertake to reduce the health and economic burdens of NCDs [1,13]. Foremost among NCDs prevention policies are the use of taxation to discourage the consumption of unhealthy products. Tax structure, consumption behavior, and enforcement capacity are important factors in the success of commodity taxes to reduce consumption and improve health. Within the health system, the most cost-effective prevention activities include counseling and advice to reduce NCD risk, especially for high-risk patients. The Collection includes articles that examine multiple aspects of taxing unhealthy products.

Nargis et al. examine patterns of tobacco cessation in different socio-economic groups in eight LMICs using the Global Adult Tobacco Surveys (GATS) (2008–2011) and International Tobacco Control Surveys (2009–2013) [14]. They use a random effects meta-analysis to estimate the odds ratios of tobacco cessation by household income, education, employment, and rural-urban residence. The study broadens the understanding of tobacco control by examining how different members of the population are affected. Distributional impacts of tobacco taxes have been extensively reviewed and recent literature has shown that–contrary to earlier conclusions–tobacco taxation is not necessarily regressive [15–18]. This study shows variation in quit rates by socio-economic status, informing the debate about tobacco tax regressivity.

Azomahou et al. examined how an increase in different types of tobacco taxes could impact the price, demand, and excise tax revenue in Senegal and Nigeria [19]. The authors first used a theoretical tax model to determine the optimal tobacco tax structure for Nigeria and Senegal, and then applied a simulation model to estimate the impact of increased tobacco taxes on price, demand, and tax revenue in the two countries. Using data from the GATS for those two countries, the study explored market share effects across different brands in an imperfectly competitive marketplace. Results indicated that Senegal would benefit from specific tobacco excise taxes whereas Nigeria would benefit more from raising ad valorem excise taxes. Increasing tobacco taxes in Senegal would reduce the public’s demand for tobacco and would decrease the country’s tax revenues. Due to a lower price elasticity of demand in Nigeria, increasing taxes would not as drastically change demand, thus resulting in a sharper increase in tax revenue.

Alcohol is another product that can be taxed to reduce consumption and related NCD risk. Shang et al. assessed the impact of different tax structures on the consumption and price variability of beer, wine, and liquor across states in the US [20]. Ordinary Least Squares regressions were performed to assess the associations between excise tax structures and price variability, for beer, wine, and liquor. Starting from the premise that more complex tax structures provide greater opportunity for strategic avoidance by consumers and price differentiation by producers, Shang et al. use price data from the Economist Intelligence Unit (EIU) and the National Institute on Alcohol Abuse and Alcoholism’s (NIAAA) policy information about state-level pricing to analyze price variability of alcohol products under differing tax regimes. Findings suggest that increased alcohol price variability can result from a mixed excise tax structure. This may reduce the effectiveness of alcohol taxation in a jurisdiction.

A key topic in the economic analysis of tax impacts on health outcomes is the elasticity of demand for products linked to raising NCD risk. Elasticity refers to the sensitivity of consumer demand to increases in factors such as price, tax or income. Elasticities vary across countries and sub-populations so their estimation can be particularly useful when derived from local settings. Araya and Paraje estimated the price and expenditure elasticities of demand for beer, wine, and liquor in Chile using the Family Budget Survey 2011–2012 and the Almost Ideal Demand System method [21]. The findings imply that consumers have many options to switch products, especially based on product quality, suggesting opportunities for tailored taxation strategies.

Chacon et al. estimated the price, expenditure, quality, and cross-price elasticity of beverage demand using household survey data from Guatemala [22]. Deaton’s Almost Ideal Demand System was used to estimate own-price, expenditure, quality, and cross-price elasticities of milk, soft drinks, packaged juices, and bottled water, controlling for the quality of the beverage. Data were obtained from the 2014 Guatemala Living Conditions National Survey. Price elasticities of demand were found to be large and negative, especially for soft drinks. The researchers inferred that a tax on sugar-sweetened beverages could be successful in reducing caloric intake with positive effects on Guatemala’s high obesity rate.

He et al. examined tobacco consumption from 2001 to 2014 across 64 countries through a novel elasticity measure, the “affordability elasticity of demand,” defined as the percentage of per capita income needed to purchase lowest-price cigarettes [23]. The findings suggest that cigarettes have become more affordable over time in lower-income countries and less affordable in higher-income countries, implying that greater increases in taxes in higher-income than in lower-income countries could be the reason. Ordinary least square regressions were used to analyze the association between cigarette affordability and consumption.

Blecher et al. measured the affordability of beer and trends in the price of beer in 92 countries from 1990 to 2016 [24]. Authors used the Relative Income Price (RIP), which uses per capita GDP, to measure the affordability and trends in the price of beer. Price data are drawn from the “Worldwide Cost of Living Survey” of the Economist Intelligence Unit (EIU), which is conducted every six months (every June and December) to assess the prices of goods and services in the world’s major cities. They found that beer is similarly priced in high-, middle-, and low-income countries, but is significantly more affordable in high-income countries. Over the 25-year period, beer has become cheaper in 49% of high-income countries and 43% of LMICs.

## Conclusions

The studies in this Special Collection provide an array of findings about the economic implications of NCDs and their risk factors across the globe. The modeling exercises point to the high social and economic costs of NCDs in a variety of contexts. The diversity of modeling approaches and outcome measures helps to provide a high level of confidence about the substantial magnitude of NCD economic impacts. Two of the studies examine NCD control programs under specific conditions in Turkey and Kenya and two studies from Bangladesh show that households affected by NCDs or tobacco use tend to allocate resources differently than other households with detrimental effects on well-being. These country studies illustrate the need to account for heterogeneity across households and health systems. Similarly, articles that analyze demand for unhealthy products in LMICs reveal new insights about how tax policy can influence health outcomes. However, there is a persistent challenge in finding adequate data in LMICs to analyze the impacts of NCDs on countries and households. More studies from LMICs focused on analyzing NCD risk factors than on assessing NCD care from the health system perspective, reflecting insufficient information on healthcare utilization in LMICs.

Many economic questions remain about the prevention and management of NCDs in LMICs. Evolving economic, demographic and nutritional changes present opportunity for health system strengthening that can address disease but additional policy regulation is needed to reduce the pervasive influence of commercially-driven health risks. Future study areas can emphasize research on guidance about how countries could optimally respond to NCDs, including employing tax-based, regulatory and enforcement tools. Many LMICs now have NCD strategies and include NCD interventions in their plans for expanding population health coverage. To determine optimal approaches for implementing these strategies, more country-level information is needed about the costs and cost-effectiveness of delivering quality care, financing NCD care, and structuring health service resources for NCD care.

## Supporting information

S1 Appendix (Table A) List of Papers in the PLOS Special Collection of Economic Cases for NCD Prevention and Control: A Global Perspective. (DOCX)

## References

[1] Bertram MY, Sweeny K, Lauer JA, Chisholm D, Sheehan P, Rasmussen B, et al Investing in non-communicable diseases: an estimation of the return on investment for prevention and treatment services. Lancet. 2018;391(10134):2071–8. 10.1016/S0140-6736(18)30665-2 29627159

[2] RTI International. The Investment Case for Non-Communicable Disease Prevention and Control in Jamaica. PAHO; 2017.

[3] Murray J, Bloch M. Non-communicable Disease Prevention and Control: A Guidance Note for Investment Cases. WHO and UNDP; 2019.

[4] Chen S, Kuhn M, Prettner K, Bloom DE. The macroeconomic burden of noncommunicable diseases in the United States: Estimates and projections. PLOS ONE. 2018;13(11):e0206702 10.1371/journal.pone.0206702 30383802 PMC6211719

[5] Hutchinson B, Small R, Acquah K, Sandoval R, Nugent R, Belausteguigoitia DI, et al The investment case as a mechanism for addressing the NCD burden: Evaluating the NCD institutional context in Jamaica, and the return on investment of select interventions. PLoS ONE. 2019; 14(10): e0223412 10.1371/journal.pone.0223412 31584979 PMC6777795

[6] Cecchini M. Use of healthcare services and expenditure in the US in 2025: The effect of obesity and morbid obesity. PLOS ONE. 2018;13(11):e0206703 10.1371/journal.pone.0206703 30403716 PMC6221341

[7] Yurekli AA, Bilir N, Husain MJ. Projecting Burden of Hypertension and its Management in Turkey. PLOS One. 2019; 14(9): e0221556 10.1371/journal.pone.0221556 31509548 PMC6738591

[8] Camacho S, Maldonado N, Bustamante J, Llorente B, Cueto E, Cardona F, et al How much for a broken heart? Costs of cardiovascular disease in Colombia using a person-based approach. PLOS ONE. 2018;13(12):e0208513 10.1371/journal.pone.0208513 30566516 PMC6300287

[9] Subramanian S, Hilscher R, Gakunga R, Munoz B, Ogola E. Cost-effectiveness of risk stratified medication management for reducing premature cardiovascular mortality in Kenya. PLOS ONE. 2019;14(6):e0218256 10.1371/journal.pone.0218256 31237910 PMC6592597

[10] Feigl AB, Goryakin Y, Devaux M, Lerouge A, Vuik S, Cecchini M. The short-term effect of BMI, alcohol use, and related chronic conditions on labour market outcomes: A time-lag panel analysis utilizing European SHARE dataset. PLOS ONE. 2019;14(3):e0211940 10.1371/journal.pone.0211940 30856184 PMC6411140

[11] Husain MJ, Datta BK, Virk-Baker MK, Parascandola M, Khondker BH. The crowding-out effect of tobacco expenditure on household spending patterns in Bangladesh. PLOS ONE. 2018;13(10):e0205120 10.1371/journal.pone.0205120 30300368 PMC6177150

[12] Datta BK, Husain MJ, Fatehin S, Kostova D. Consumption displacement in households with noncommunicable diseases in Bangladesh. PLOS ONE. 2018;13(12):e0208504 10.1371/journal.pone.0208504 30543648 PMC6292644

[13] Jamison DT, Alwan A, Mock CN, Nugent R, Watkins D, Adeyi O, et al Universal health coverage and intersectoral action for health: key messages from Disease Control Priorities, 3rd edition. Lancet. 2017. 10.1016/S0140-6736(17)32906-9 PMC5996988 29179954

[14] Nargis N, Yong H-H, Driezen P, Fong GT, Thompson ME, Boland R, et al Socioeconomic patterns of smoking cessation behavior in low and middle-income countries: Emerging evidence from the Global Adult Tobacco Surveys and International Tobacco Control Surveys. PLOS ONE. 2019; 14(9): e0220223 10.1371/journal.pone.0220223 31490958 PMC6730869

[15] Remler DK. Poor smokers, poor quitters, and cigarette tax regressivity. American journal of public health. 2004;94(2):225–9. 10.2105/ajph.94.2.225 14759931 PMC1448232

[16] Verguet S, Gauvreau CL, Mishra S, MacLennan M, Murphy SM, Brouwer ED, et al The consequences of tobacco tax on household health and finances in rich and poor smokers in China: an extended cost-effectiveness analysis. Lancet Glob Health. 2015;3(4):e206–16. 10.1016/S2214-109X(15)70095-1 25772692

[17] Chaloupka FJ, Yurekli A, Fong GT. Tobacco taxes as a tobacco control strategy. Tobacco Control. 2012;21(2):172–80. 10.1136/tobaccocontrol-2011-050417 22345242

[18] Chaloupka FJ, Warner KE. The Economics of Smoking. National Bureau of Economic Research Working Paper Series. 1999;No. 7047.

[19] Azomahou TT, Baldé R, Diagne A, Mané PY, Kaba IS. Public finances and tobacco taxation with product variety: Theory and application to Senegal and Nigeria. PLOS ONE. 2019;14(2):e0212015 10.1371/journal.pone.0212015 30763374 PMC6375595

[20] Shang C, Wang X, Chaloupka FJ. The association between excise tax structures and the price variability of alcoholic beverages in the United States. PLOS ONE. 2018;13(12):e0208509 10.1371/journal.pone.0208509 30589849 PMC6307826

[21] Araya D, Paraje G. The impact of prices on alcoholic beverage consumption in Chile. PLOS ONE. 2018;13(10):e0205932 10.1371/journal.pone.0205932 30346989 PMC6197648

[22] Chacon V, Paraje G, Barnoya J, Chaloupka FJ. Own-price, cross-price, and expenditure elasticities on sugar-sweetened beverages in Guatemala. PLOS ONE. 2018;13(10):e0205931 10.1371/journal.pone.0205931 30346999 PMC6197849

[23] He Y, Shang C, Chaloupka FJ. The association between cigarette affordability and consumption: An update. PLOS ONE. 2018;13(12):e0200665 10.1371/journal.pone.0200665 30517093 PMC6281249

[24] Blecher E, Liber A, Van Walbeek C, Rossouw L. An international analysis of the price and affordability of beer. PLOS ONE. 2018;13(12):e0208831 10.1371/journal.pone.0208831 30557353 PMC6296500

