# Correction: Introducing the PLOS special collection of economic cases for NCD prevention and control: A global perspective

**DOI:** 10.1371/journal.pone.0233141

**Published:** 2020-05-07

**Authors:** Rachel A. Nugent, Muhammad Jami Husain, Deliana Kostova, Frank Chaloupka

There are errors in the Funding statement. The correct Funding statement is as follows: This Collection was supported by TEPHINET, a program of The Task Force for Global Health, Inc., via Cooperative Agreement number NU2GGH001873, funded by the Centers for Disease Control and Prevention. Its contents are solely the responsibility of the authors and do not necessarily represent the official views of the Centers for Disease Control and Prevention, the U.S. Department of Health and Human Services, The Task Force for Global Health, Inc., or TEPHINET.
