# Supplementary material for: Introducing the PLOS special collection of economic cases for NCD prevention and control: A global perspective
Source: PLoS One. 2020 Feb 6;15(2):e0228564. doi: 10.1371/journal.pone.0228564 (PMC7004318; doi:10.1371/journal.pone.0228564)
Supplement: S1 Appendix — (DOCX) [file pone.0228564.s001.docx]

S1 Appendix: (Table A) List of Papers in the PLOS Special Collection of Economic Cases for NCD Prevention and Control: A Global Perspective.

*Table A1: List of Papers in the PLOS Special Collection of Economic Cases for NCD Prevention and Control: A Global Perspective**

| Citation (APA) | Main research question/objective | Main research result(s) | Country/Time period/Population | Analysis method | Data Sources |
| --- | --- | --- | --- | --- | --- |
| National analyses including NCD investment cases | | | | | |
| Chen S, Kuhn M, Prettner K, Bloom DE (2018) The macroeconomic burden of noncommunicable diseases in the United States: Estimates and projections. PLoS ONE 13(11):e0206702.  <https://doi.org/10.1371/journal.pone.0206702> | Assess the extent to which NCDs will affect US productive capacity in 2015-2050 and calculate the resulting economic burden in terms of foregone GDP. | • Total loss of USD 94.9 trillion due to all NCDs, or USD 265,000 per capita. Roughly corresponds to an annual tax rate of 10.8% on aggregate income. • Mental health conditions and cardiovascular diseases impose the highest burdens, followed by cancer, diabetes, and chronic respiratory diseases. | U.S./2015-2050/ U.S., all ages, male and female, all ethnicities, all socio-economic status | Macroeconomic framework incorporating endogenous adjustment mechanisms | World Bank; Global Burden of Disease Study; International Labor Organization; Barro-Lee education database; Penn World Tables. |
| Hutchinson B, Small R, Acquah K, Sandoval R, Nugent R, Belausteguigoitia DI, et al. (2019) The investment case as a mechanism for addressing the NCD burden: Evaluating the NCD institutional context in Jamaica, and the return on investment of select interventions. PLoS ONE 14(10): e0223412.  <https://doi.org/10.1371/journal.pone.0223412> | Examine “investment cases” as a potential mechanism for catalyzing attention to, and funding for, NCDs. Conduct an economic analysis to estimate the return-on-investment from scaling up strategic clinical interventions and policy measures in Jamaica. | • Scaling up clinical interventions that target CVD, diabetes, and mental health disorders, and policy measures that target tobacco and alcohol use, would save over 6,600 lives between 2017-2032, and avert JMD 81.3 billion (USD 640 million) in direct and indirect economic costs.  • Use of results to advocate for increased funding and targeted action indicates promise for investment cases to spur change that can reduce NCD burdens. | Jamaica/2017-2032/population model | Institutional and context analysis (ICA), return-on-investment economic analysis using the UN Inter-Agency OneHealth Tool | NCD Impact Module of the Inter-UN Agency OneHealth Tool; Technical Briefs in Appendix 3 of the WHO NCD Action Plan; WHO NCD Costing Tool; local government and NGO sources; stakeholder interviews. |
| Cecchini M (2018) Use of healthcare services and expenditure in the US in 2025: The effect of obesity and morbid obesity. PLoS ONE 13(11): e0206703.  <https://doi.org/10.1371/journal.pone.0206703> | Assess the contribution of BMI categories in shaping past trends of use of healthcare services and associated expenditure in the US and project results to 2025. | • 20.5 million individuals will be severely obese in 2025.  • Normal-weight and overweight individuals have stable trends in healthcare use but will increase substantially for patients in class II and class III obesity. • Total healthcare expenditure increases more quickly in the obese population than in normal-weight individuals. | US/2000-2025/ US, all ages, male and female, all ethnicities, all socio-economic status | Logistic regression to model the probability of use of healthcare service; generalized linear model, conditional on having positive access to the healthcare service. | Medical Expenditure Panel Survey (MEPS); National Health and Nutrition Examination Survey; US Census Bureau. |
| Yurekli AA, Bilir N, Husain MJ (2019) Projecting burden of hypertension and its management in Turkey, 2015-2030. PLoS ONE 14(9): e0221556.  <https://doi.org/10.1371/journal.pone.0221556> | Apply existing research finding regarding the impact of a population-wide reduction in sodium consumption on the decrease of the hypertension prevalence rate among 15+ years population and the gender-age specific reduction in total death rates among 30+ years population, and compare hypertension burden, averted deaths, costs and benefits between two scenarios. | • Under the status quo, about 55% of hypertensive people will be under the treatment, but that would increase to 74% under Scenario II.  • 24,300 deaths and 392,000 DALYs will be averted by 2030.  • The economic benefits of reduced hypertension deaths are estimated to be 6.7 to 8.6 times higher than the additional cost of hypertension treatment. | Turkey/2015-2030/ National | Projections of prevalence and DALYs lost to hypertension are made and converted to economic terms. Costs are estimated based on pharmacy prices and existing treatment patterns. | Chronic Diseases Risk Factor (CDRFS) survey, 2011; Global Burden of Disease Study; World Bank; Turkish Statistical Institute; medication prices gathered locally. |
| Camacho S, Maldonado N, Bustamante J, Llorente B, Cueto E, Cardona F, et al. (2018) How much for a broken heart? Costs of cardiovascular disease in Colombia using a person-based approach. PLoS ONE 13(12): e0208513.  <https://doi.org/10.1371/journal.pone.0208513> | Assess the healthcare costs of cardiovascular disease (coronary heart disease and stroke) in Colombia, using a person-based approach. | • The average annual healthcare cost for a person with coronary heart disease in Colombia ranges from INT$ 4,277 and INT$ 4,846. • The average annual healthcare cost for a person with stroke ranges from INT$ 5,816 and INT$ 6,616. | Colombia/2010-2012/ Colombian adults over 30 years of age who fall into the contributory regime for health insurance. | Calculate the annual healthcare cost of an individual who has been diagnosed with CVD. | The Colombian Association of Private Health Insurance Companies of the Contributory Regime (ACEMI); The Enrolled Population dataset. |
| Subramanian S, Hilscher R, Gakunga R, Munoz B, Ogola E (2019) Cost-effectiveness of risk stratified medication management for reducing premature cardiovascular mortality in Kenya. PLoS ONE 14(6): e0218256.  <https://doi.org/10.1371/journal.pone.0218256> | Assess the cost-effectiveness of a risk-stratified approach to medication management in Kenya in order to achieve adequate blood pressure control to reduce CVD events. | • Treating high-risk individuals only was more cost-effective than treating those with both high and moderate CVD risk.  • Medication management was only cost-effective under the low-cost scenario.  • The incremental cost per DALY gained in the low-cost scenario was under the $4,785 (3 times GPD per capita) threshold for Kenya.  • Under the low-cost scenario, even lower levels of effectiveness of medication management are likely to be cost-effective for high-risk men and women. | Kenya/2015/ Kenyan men and women aged 25-69 | Microsimulation model to evaluate CVD risk over the lifetime of a cohort of individuals | Kenya STEPwise survey for non communicable diseases risk factors 2015 report. |
| Feigl AB, Goryakin Y, Devaux M, Lerouge  A, Vuik S, Cecchini M (2019) The short-term effect of BMI, alcohol use, and related chronic conditions on labour market outcomes: A time-lag panel analysis utilizing European SHARE dataset. PLoS ONE 14(3): e0211940.  <https://doi.org/10.1371/journal.pone.0211940> | Assess the longitudinal impact of alcohol use and high BMI on labour market outcomes in the European region by modeling the direct effect of high BMI and alcohol use, and the effect via associated diseases. | • Controlling for other chronic conditions, being overweight increases employment likelihood among men, but not among women.  • Obesity decreased female, but not male, employment chances.  • All chronic conditions linked with high BMI decreased employment likelihood and significantly increased intent to retire early. • Alcohol use positively affects employment likelihood in women at all drinking levels relative to lifetime abstainers, but only for moderate (not heavy) drinkers. | EU member states, plus Norway, Iceland, and Switzerland/2004-2007, 2010-2013, and 2015 / Adults aged 50–63 years, male and female | The impact of BMI, alcohol use, and associated diseases on several outcomes were modelled via lagged Poisson and Zero-inflated Poisson regressions, adjusting for missingness via inverse probability weighting. | SHARE (Survey of Health, Ageing and Retirement in Europe) Release 6.0. |
| NCD Impacts on Household Resource Allocation | | | | | |
| Husain MJ, Datta BK, Virk-Baker MK, Parascandola M, Khondker BH (2018) The crowding-out effect of tobacco expenditure on household spending patterns in Bangladesh. PLoS ONE 13(10): e0205120.  <https://doi.org/10.1371/journal.pone.0205120> | Assess the crowding-out effect of tobacco consumption in Bangladesh. | • Tobacco user households allocated less to clothing, housing, education, energy, and transportation and communication.  • Expenditures on food and healthcare of tobacco user households are greater than those of non-user households. | Bangladesh/2010/ Bangladesh households in rural and urban areas, nationally-representative | Comparison of household expenditures among household with and without tobacco smokers using seemingly unrelated regression models | Bangladesh Household Income and Expenditure Survey 2010. |
| Datta BK, Husain MJ, Fatehin S, Kostova D (2018) Consumption displacement in households with noncommunicable diseases in Bangladesh. PLoS ONE 13(12): e0208504.  <https://doi.org/10.1371/journal.pone.0208504> | Assess the role of NCDs in household resource allocation in Bangladesh. | • Presence of NCDs in the household was associated with lower relative expenditure on clothing and housing in all economic subgroups, and with lower expenditure on food among marginally poor households.  • Presence of NCDs was associated with higher household spending on tobacco and higher-calorie foods and with lower spending on fish, vegetables, and legumes.  • Medical expenditure share was 59% higher for NCD households. | Bangladesh/2010/ Bangladesh households in rural and urban areas, nationally-representative | Comparison of household expenditure allocations between NCD and non-NCD households using regression models. | Bangladesh Household Income and Expenditure Survey 2010. |
| Risk Factor Policies | | | | | |
| Nargis N, Yong H-H, Driezen P, Mbulo L, Zhao L, Fong GT, et al. (2019) Socioeconomic patterns of smoking cessation behavior in low and middle-income countries: Emerging evidence from the Global Adult Tobacco Surveys and International Tobacco Control Surveys. PLoS ONE 14(9):e0220223.  <https://doi.org/10.1371/journal.pone.0220223> | Examine the association between smoking cessation behaviors and socio-economic status (SES) of smokers from eight low- and middle-income countries. | • No clear evidence of an association between SES indicators and successful quitting, except for employed smokers, who were less likely to quit than their non-employed counterparts. | Bangladesh, Brazil, China, India, Malaysia, Mexico, Thailand, Uruguay/2008-2013/nationally representative household survey | Adjusted odds ratios (AORs) of successful quitting, multivariable logistic regression, and random effects meta-analysis to combine estimates of AORs | International Tobacco Control (ITC) Surveys; Global Adult Tobacco Surveys (GATS) |
| Azomahou TT, Balde´ R, Diagne A, Mane´  PY, Kaba IS (2019) Public finances and tobacco taxation with product variety: Theory and application to Senegal and Nigeria. PLoS ONE 14(2): e0212015.  <https://doi.org/10.1371/journal.pone.0212015> | • Assess which category of excise tobacco taxes is more appropriate for Senegal and Nigeria  • Model effects of tobacco tax on the price, demand, and tax revenue in Senegal and Nigeria | • Specific excise taxes are more adapted to Senegal while ad valorem excise taxes best fit Nigeria. • Increasing taxes in Senegal strongly reduces the demand but may induce a decrease in the tax revenues. • Increasing taxes in Nigeria will lead to a lesser decline in demand, but will result in a sharp increase of the country’s tax revenues. | Senegal and Nigeria/ Senegal - 2015 Nigeria – 2012/ Senegal and Nigeria, tobacco users and policy makers | Theoretical and simulation models of taxation. | World Health Organization’s Global Adult Tobacco Survey (GATS) for Senegal and Nigeria. |
| Shang C, Wang X, Chaloupka FJ (2018) The association between excise tax structures and the price variability of alcoholic beverages in the United States. PLoS ONE 13(12): e0208509.  <https://doi.org/10.1371/journal.pone.0208509> | Estimate links between excise tax structure and alcohol consumption through price variability | • Compared with a specific excise tax, a mixed structure with both specific and ad valorem components was associated with 38% greater beer price variability.  • A mixed excise tax structure for liquor was associated with 60–77% greater liquor price variability.  • These associations do not imply a causal link between tax structures and price variability. | US/2003-2016/ US alcohol consumers and policy makers | Ordinary Least Squares regressions were used to assess the associations between excise tax structures and price variability, for beer, wine, and liquor. | Economist Intelligence Unit city data; The National Institute on Alcohol Abuse and Alcoholism’s (NIAAA) Alcohol Policy Information System. |
| Araya D, Paraje G (2018) The impact of prices on alcoholic beverage consumption in Chile. PLoS ONE 13(10): e0205932.  <https://doi.org/10.1371/journal.pone.0205932> | Estimate the demand elasticities (own-price, cross-price, expenditure and quality) for three groups of alcoholic beverages in Chile: spirits, wines, and beers. | • The estimated demand elasticities were more inelastic for spirits, followed by wines and beers.  • Demand for spirits was less sensitive to changes in the total budget, while demand for wines was most sensitive. • Wines also reported the most sensitivity related to quality for changes in the total budget. | Chile/2011-2012/ Chile alcohol consumers and policy makers | Demand elasticities were obtained using the Almost Ideal Demand System (AIDS) modified to adjust for household preferences by demographic characteristics. | VII Encuesta de Presupuestos Familiares (Family Budget Survey) 2011–2012 conducted by the National Institute of Statistics. |
| Chacon V, Paraje G, Barnoya J, Chaloupka FJ (2018) Own-price, cross-price, and expenditure elasticities on sugar-sweetened beverages in Guatemala. PLoS ONE 13(10): e0205931.  <https://doi.org/10.1371/journal.pone.0205931> | Estimate the price, expenditure, quality, and cross-price elasticity of beverage demand using household survey data. | • Positive expenditure on soft drinks was the highest of the different beverages examined; bottled water was next highest for urban households but lowest for rural households.  • Own-price elasticities for all beverages were negative and statistically significant.  • The expenditure elasticity for soft drinks suggests that a 10% expenditure increase would result in a 9.9% increase in demand.  • Milk and soft drinks have positive quality elasticity. | Guatemala/2014/ Guatemala public and policy makers | Estimations perfomed using Deaton’s Almost Ideal Demand System. | 2014 Guatemala Living Conditions National Survey (ENCOVI). |
| He Y, Shang C, Chaloupka FJ (2018) The association between cigarette affordability and consumption: An update. PLoS ONE 13(12): e0200665.  <https://doi.org/10.1371/journal.pone.0200665> | Examine the association between cigarette affordability and cigarette consumption among 78 countries and calculate the affordability elasticity of demand. | • From 2001 to 2014, cigarette consumption rose in low-income countries and decreased slightly in lower middle-income countries. The Relative Income Price of cigarettes declined in low- and lower middle-income economies.  • Real cigarette prices declined in low- and lower middle-income countries and rose in upper middle- and high-income countries. Cigarettes were more affordable in HICs than in LMICs. • A 10% increase in the Relative Income Price of cigarettes was associated with a 2% decrease in per capita consumption. | 78 countries worldwide/2001-2014/ Price of cigarettes globally | Relative Income Price (RIP) ratio and ordinary least square regressions | World Bank World Development Indicator (WDI) database; Economist Intelligence Unit; EuroMonitor International; Framework Convention on Tobacco Control. |
| Blecher E, Liber A, Van Walbeek C, Rossouw L (2018) An international analysis of the price and affordability of beer. PLoS ONE 13(12): e0208831.  <https://doi.org/10.1371/journal.pone.0208831> | Measure the affordability of beer in a large cross-section of countries and investigate trends in beer affordability over time. | • While beer is similarly priced in high-income and LMICs, it is significantly more affordable in HICs.  • There is significant variation in both price and affordability in HICs and in LMICs. Beer has become cheaper in real terms in 49% of HICs and 43% of LMICs. Beer became more affordable in most HICs and LMICs. | 92 countries worldwide/1990-2016/ Price of beer globally | Relative Income Price (RIP) | Worldwide Cost of Living Survey of the Economist Intelligence Unit (EIU). |

**Website link for the Special Collection:* [*https://collections.plos.org/ncds-economics*](https://collections.plos.org/ncds-economics)
